# RAB37 Promotes Adipogenic Differentiation of hADSCs via the TIMP1/CD63/Integrin Signaling Pathway

**DOI:** 10.1155/2021/8297063

**Published:** 2021-11-23

**Authors:** Haili Huang, Anran Li, Jin Li, Dan Sun, Ling Liang, Kunyan Pan, Chengzhang He, Peihua Zhang

**Affiliations:** Department of Plastic Surgery, Affiliated Hospital of Guangdong Medical University, China

## Abstract

The adipogenic differentiation ability of human adipose-derived mesenchymal stem cells (hADSCs) is critical for the construction of tissue engineering adipose, which shows promising applications in plastic surgery and regenerative medicine. RAB37 is a member of the small RabGTPase family and plays a critical role in vesicle trafficking. However, the role of RAB37 in adipogenic differentiation of hADSCs remains unclear. Here, we report that both the mRNA and protein levels of RAB37 fluctuated during adipogenic differentiation. Upregulation of RAB37 was observed at the early stage of adipogenic differentiation, which was accompanied by increased expression of transcription factors PPAR*γ*2 and C/EBP*α*, and lipoprotein lipase (LPL). Overexpression of RAB37 promoted adipogenesis of hADSCs, as revealed by Oil Red O staining and increased expression of PPAR*γ*2, C/EBP*α*, and LPL. Several upregulated cytokines related to RAB37-mediated adipogenic differentiation were identified using a cytokine array, including tissue inhibitor of matrix metalloproteinase 1 (TIMP1). ELISA confirmed that upregulation of RAB37 increased the secretion of TIMP1 by hADSCs. Proximity ligation assay showed that RAB37 interacts with TIMP1 directly. Knockdown of TIMP1 compromised RAB37-mediated adipogenic differentiation. In addition, TIMP1 binds membrane receptor CD63 and integrin *β*1. RAB37 promotes Tyr397 phosphorylation of FAK, an important protein kinase of the integrin *β*1 signaling. Moreover, both knockdown of CD63 and inhibitor of FAK impeded RAB37-mediated adipogenic differentiation. In conclusion, RAB37 positively regulates adipogenic differentiation of hADSCs via the TIMP1/CD63/integrin *β*1 signaling pathway.

## 1. Introduction

The primary and secondary soft tissue defects are a common problem in orthopedic surgery. Autologous fat, as a kind of soft tissue filler, can be applied in facial rejuvenation, chest shaping, filling of soft tissue depression in various parts of the body, and prevention and treatment of wounds and scars (1–3). However, the clinical effects of fat transplantation are not stable. The survival rate of autologous fat is the key factor limiting the effects of autologous fat transplantation. Recent studies have demonstrated that ADSCs may assist autologous fat transplantation to repair primary and secondary soft tissue defects with significant efficacy and few complications (4). The survival rate of autologous fat cells with ADSCs was about 70% (4). On the one hand, ADSCs can differentiate into adipocytes (5, 6). On the other hand, they can secrete cytokines to improve the survival rate of autologous fat (5, 6). However, there are few studies focusing on the regulatory mechanisms of adipogenic differentiation in ADSCs.

Regulation of adipogenic differentiation is a complicated process requiring multiple regulators and sophisticated regulatory networks (7–10). hADSCs secrete a lot of cytokines that are involved in proliferation and differentiation of stem cells (11). Consequentially, regulators of cytokine secretion also affect proliferation and differentiation. RabGTPases are critical regulators of vesicle trafficking. They participate in various cellular processes and diseases by controlling vesicle trafficking of critical proteins. Several RabGTPases have been proved to be involved in lipid metabolism and homeostasis (12, 13). RAB37 is a member of RabGTPases first identified in mast cell degranulation (14). It also plays regulatory roles in lung cancer by controlling secretion of several cytokines that are involved in the regulation of tumor growth (15, 16), metastasis (15, 16), and cancer stem cell renewal (17). We screened differentially expressed RabGTPases in ADSCs after induction of adipogenic differentiation. Increased expression of RAB37 was observed after induction of adipogenic differentiation.

Tissue inhibitor of matrix metalloproteinase 1 (TIMP1) is one of the dominant inhibitor of matrix metalloproteinases (MMPs). The abundance of TIMP1 in the secretome of adipose-derived stem cells has been demonstrated by various studies (11, 18). TIMP1 is a multifunctional cytokine. In addition to its inhibitory effects on MMPs, TIMP1 also exhibits antiapoptosis effects and accelerates cell growth (19). We previously showed that TIMP1 promotes the proliferation of hADSCs (20). A previous study in obesity demonstrates that TIMP1 modulates adipogenesis (21). The multifunctional roles of TIMP1 in various cellular activities require the binding of TIMP1 to membrane receptor CD63 and integrin *β*1, which activates the integrin *β*1 signaling pathway (22, 23).

In the current study, we investigated the role of RAB37 in adipogenic differentiation of hADSCs and identified TIMP1 as a cytokine associated with RAB37-mediated adipogenic differentiation.

## 2. Materials and Methods

### 2.1. Isolation and Characterization of hADSCs

Samples of human adipose tissues were obtained by lipoaspiration or biopsy from abdominal subcutaneous fat and then processed for the isolation and culture of human adipose-derived mesenchymal stem cells (hADSCs) as previously described (20). The human adipose tissue collection was approved by the Institutional Review Board of Affiliated Hospital of Guangdong Medical University. Written informed consent was obtained from all patients prior to sample collection. The multipotency of hADSCs was confirmed by adipogenic, osteogenic, and endothelial differentiation as previously described (20). Adipogenic and osteogenic differentiations were evaluated by Oil Red O staining (Sigma-Aldrich, St. Louis, MO, USA) for lipid droplets and Alizarin red staining (Sigma-Aldrich, St. Louis, MO, USA) for calcium deposit. Endothelial differentiation was measured by tube formation assays.

### 2.2. Adipogenic Differentiation of hADSCs In Vitro

At passage 4, the cells were seeded on collagen type I-coated culture dishes at a density of 2.0 × 10^4^ cells/cm^2^. When the cells reached 100% confluence, adipogenic induction was carried out over a period of 14 days as previously described (20). Adipogenic differentiation was evaluated by Oil Red O staining (Sigma-Aldrich, St. Louis, MO, USA).

### 2.3. Oil Red O Staining

Oil Red O staining was performed as previously described (20). In brief, cells were fixed with 4% paraformaldehyde for 15 min at room temperature, incubated with propylene glycol for 2 min, stained with Oil Red O solution for 50 min, differentiated with 85% propylene glycol for 1 min, and then incubated with hematoxylin for 1 min. Cells were visualized using a Leica microscope (Leica, Germany).

### 2.4. Quantitative Real-Time PCR (qRT-PCR)

Total RNA was extracted from tissues or cells using TRIzol reagent (Invitrogen). Complementary DNA (cDNA) was reversely transcribed from RNA using a reverse transcriptase kit (TAKARA, Japan). qRT-PCR was performed using the FastStart Universal SYBR Green Master mix (TAKARA, Japan) on a Roche Thermal Cycler (Lightcycler 480 II, Roche, Switzerland). GAPDH was used as the internal control. The 2−*ΔΔ* Ct method was performed to compute the relative expression levels. The qRT-PCR primers are listed in Table S1.

### 2.5. Creation of Stable Cell Line of RAB37

The lentiviruses expressing shRNA targeting RAB37 (lv-shRNA), scramble shRNA (lv-NC), and RAB37 (lv-RAB37) and control lentivirus (lv-vector) were obtained from GeneChem (Shanghai, China). To establish hADSCs stably expressing shRNA targeting RAB37, hADSCs were infected with lentivirus lv-shRNA. The control group was infected with lv-NC. To establish hADSCs stably expressing RAB37, hADSCs were infected with lv-RAB37. The control group was infected with lv-vector. The infected cells were selected for 14 days in the presence of 2 *μ*g/mL puromycin (Sigma-Aldrich, St. Louis, MO, USA). The expression of RAB37 in infected cells was verified by qRT-PCR. hADSCs stably expressing shRNA targeting RAB37 and control shRNA were named as hADSCs-shRNA and hADSCs-NC. hADSCs stably expressing RAB37 were named as hADSCs-RAB37. The control group was named as hADSCs-vector.

### 2.6. Cytokine Array

An antibody-based cytokine array system (Human Cytokine Antibody Array G2000, RayBiotech) was used to detect the levels of cytokines and growth factors in hADSCs-RAB37 or hADSCs-vector supernatants. Cytokine array screening was performed according to the manufacturer's instruction as previously described (24). Briefly, the glass slide was blocked with blocking buffer and incubated sequentially with supernatants, Biotinylated Detection Antibody Cocktail, and Streptavidin-Conjugated Fluor. The glass slide was scanned with a gene microarray laser scanner. Densitometry analysis was performed to determine upregulated cytokines in hADSCs-RAB37 supernatants.

### 2.7. TIMP1 ELISA

To determine the secretion of human TIMP1, supernatants of hADSCs-vector or hADSCs-RAB37 were collected at different time points. The level of TIMP1 was measured by the human TIMP1 ELISA kit (Cat. no. RK00051, ABclonal, Wuhan, China) according to the manufacturer's instructions. Supernatants were diluted in a range from 10- to 100-fold to obtain values falling within the linear range of the standard curve.

### 2.8. Western Blotting

Western blotting was performed as previously described (25). Briefly, cells were harvested and lysed in RIPA lysis buffer supplemented with a protease inhibitor cocktail (Sigma-Aldrich, St. Louis, MO, USA). Some 30 *μ*g of proteins were separated by 10% or 12% SDS-PAGE and then transferred to PVDF membranes (Millipore, USA). After blocking in 5% nonfat milk, the membranes were incubated with the primary antibodies. The anti-RAB37 antibody (ab67267) and anti-lipoprotein lipase antibody (ab21356) were obtained from Abcam. The anti-PPAR*γ* antibody (2435), anti-C/EBP*α* antibody (8178), anti-phospho FAK (Tyr397) antibody (8556), anti-*β*-actin antibody (3700), and anti-GAPDH antibody (5174) were obtained from Cell Signaling Technology. The proteins were visualized using enhanced chemiluminescence reagents (Cat. no. 32132, Pierce).

### 2.9. Proximity Ligation Assay (PLA)

All PLA reagents, if not otherwise stated, were purchased from Duolink (Sigma-Aldrich, St. Louis, MO, USA). PLA was performed according to manufactures' instructions. Briefly, hADSCs were fixed with 4% paraformaldehyde, incubated with PBS containing 0.1% Triton X-100, and blocked with Blocking Solution for 60 min. hADSCs were incubated with primary antibodies at 4°C overnight. Primary antibodies and their combinations used for the assays are specified in Supplementary Table S2. Unbound antibodies were removed by washing in PBS-T, and the cells were then incubated with oligonucleotide-conjugated secondary antibodies (PLA Probes) PLUS and MINUS for 1 h at 37°C. Subsequently, cells were washed and incubated with a ligation mixture (Detection Reagent Red) for 30 min at 37°C. Cells were washed and incubated with an amplification mixture (Detection Reagent Red) for 100 min at 37°C. After amplification, cells were washed and rinsed in distilled water. The nuclei were stained with DAPI. Cells were visualized using a FV3000 Olympus laser scanning confocal microscope with a 100x objective. Rolling circle products (RCPs) were quantified using the Duolink Image Tool. For clear visualization of RCPs, the PLA images were processed with ImageJ software using a maximum filter (3 pixels) for the PLA channel.

### 2.10. Statistical Analysis

Student's *t*-test was performed to analyze statistical differences. The association between relative protein amount of RAB37 and TIMP1 secretion during adipogenic differentiation using Pearson correlation analysis. The statistical graphs were made by GraphPad Prism 6.0 software. Data were shown as mean ± SD. *p* < 0.05 was considered statistically significant.

## 3. Results

### 3.1. RAB37 Fluctuated during Adipogenic Differentiation

**Figure 1:**
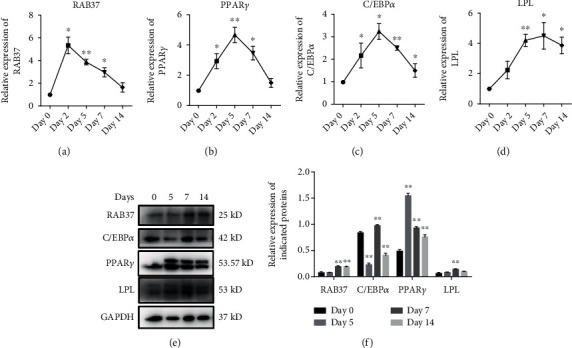
The expression of RAB37 fluctuated during adipogenic differentiation of ADSCs. Adipogenic induction of hADSCs was carried out over a period of 14 days. The expression of RAB37 (a) and adipogenesis-related genes, PPAR*γ* (b), C/EBP*α* (c), and LPL (d), was examined by qRT-PCR and western blotting (e) at the indicated time points. (f) Densitometry analysis of the western blots, *n* = 3. ^∗^*p* < 0.05; ^∗∗^*p* < 0.01.

We first examined the expression of RAB37 during adipogenic differentiation of hADSCs using qRT-PCR and western blotting. The hADSCs were isolated, and the multipotency of the hADSCs was confirmed by adipogenic, osteogenic, and endothelial differentiation (Figure S1). The adipogenic induction was carried out over a period of 14 days. The status of adipogenic differentiation was determined by the expression of PPAR*γ*, C/EBP*α*, and lipoprotein lipase (LPL). Increased mRNA expression of RAB37 was observed at day 2 of adipogenic induction and decreased since day 5. The mRNA expression of PPAR*γ* and C/EBP*α* reached the peak at day 5 and then gradually decreased. The mRNA expression of LPL gradually increased and was maintained at a high level at the late stage of adipogenic differentiation (Figures 1(a)–1(d)). The increased protein amount of RAB37 was observed since day 5 and maintained at a relatively high level until day 14. The protein amount of C/EBP*α* and LPL reached the peak at day 7, and PPAR*γ* reached the peak at day 5 (Figures 1(e) and 1(f)).

### 3.2. RAB37 Promoted Adipogenic Differentiation

**Figure 2:**
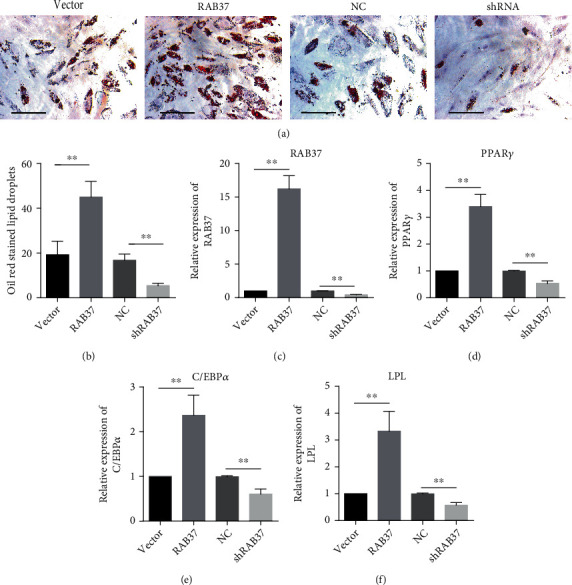
RAB37 promotes adipogenic differentiation of ADSCs. hADSCs were infected with lentivirus expressing RAB37 or shRNA targeting RAB37 (shRAB37). Empty vector lentivirus (vector) or lentivirus containing scramble RNA (NC) served as control, respectively. Oil Red O staining was used to evaluate adipogenesis of hADSCs (a) (scale bar = 300 *μ*m). Oil Red O-stained hADSCs in six random views were counted (b), *n* = 6. The expression of RAB37 (c), PPAR*γ* (d), C/EBP*α* (e), and LPL (f) were examined by qRT-PCR, *n* = 3. Data are presented as means ± SD. ^∗∗^*p* < 0.01; vector vs. RAB37, and NC vs. shRNA.

To investigate the role of RAB37 in adipogenic differentiation, lentivirus expressing RAB37 (lv-RAB37) or shRNA targeting RAB37 (shRAB37) was used to manipulate the expression of RAB37 in hADSCs. The empty vector lentivirus or lentivirus containing scramble RNA (NC) was used as control for lv-RAB37 or shRAB37, respectively. The adipogenic induction was carried out over a period of 14 days. Oil Red O staining showed increased lipid droplets in hADSCs-RAB37 and decreased lipid droplets in hADSCs-shRAB37, compared with their controls (Figures 2(a) and 2(b)). Correspondingly, increased expression of PPAR*γ*, C/EBP*α*, and LPL was observed in hADSCs-RAB37 and decreased expression was observed in hADSCs-shRAB37 (Figures 2(c)–2(f)).

### 3.3. RAB37 Promoted TIMP1 Secretion during Adipogenic Differentiation

**Figure 3:**
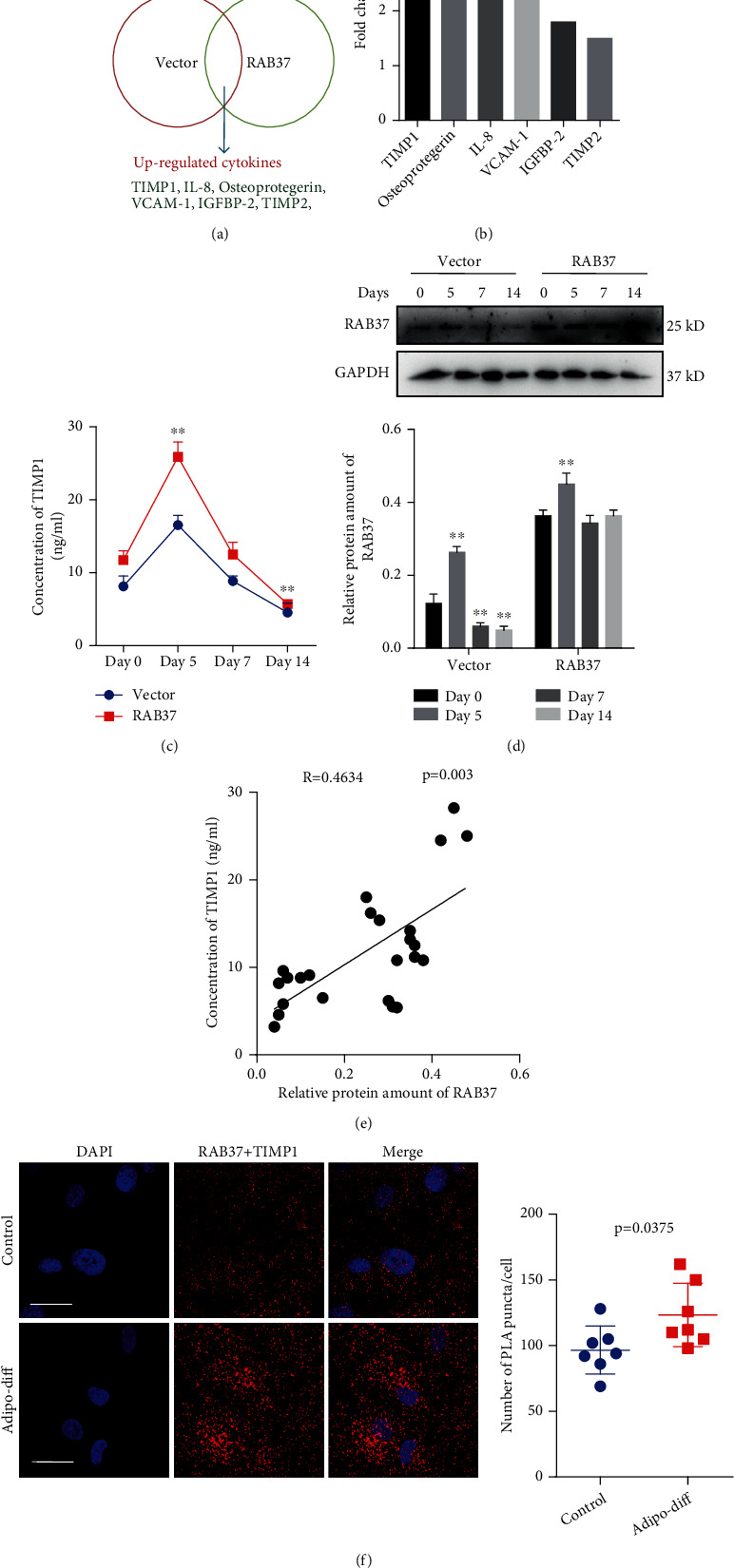
RAB37 promotes secretion of TIMP1: (a) schematic of the screening strategy for upregulated cytokines in RAB37-mediated adipogenic differentiation of hADSCs; (b) fold changes of upregulated cytokines; (c) ELISA was performed to evaluate the concentration of TIMP1 in the supernatant of hADSCs during adipogenic differentiation (*n* = 6); (d) relative protein amount of RAB37 was determined by western blotting, *n* = 3; (e) correlation analysis between the relative protein amount of RAB37 and the concentration of TIMP1 calculated using Pearson analysis; (f) proximity ligation assay (PLA) was used to examine the interaction between RAB37 and TIMP1 on day 5 of adipogenic differentiation. Scale bar = 50 *μ*m. Eight random views were counted, *n* = 8.

To explore the mechanism of RAB37-mediated differentiation, we focused on the effects of RAB37 on the secretion of cytokines. Cytokine array screening was performed to examine the differential expression of cytokines in hADSCs-RAB37 and hADSCs-vector. Our data showed increased accumulation of tissue inhibitor of matrix metalloproteinase 1 (TIMP1), osteoprotegerin, interleukin-8 (IL-8), vascular cell adhesion molecule-1 (VCAM-1), insulin-like growth factor binding protein-3 (IGFBP-3), and tissue inhibitor of matrix metalloproteinase 2 (TIMP2) in the supernatant of hADSCs-RAB37 after induction of adipogenic differentiation (Figure 3(b)). Among these molecules, TIMP1 has drawn our special attention. We previously observed that TIMP1 modulates proliferation of hADSCs (26). ELISA confirmed increased accumulation of TIMP1 in the supernatant of hADSCs-RAB37, and the concentration of TIMP1 reached the peak after 5 days of adipogenic induction (Figure 3(c)). Afterwards, an increased protein amount of RAB37 was observed at the early stage of adipogenic differentiation of both hADSCs-RAB37 and hADSCs-vector (Figure 3(d)). We explored the association between the relative protein amount of RAB37 and TIMP1 secretion during adipogenic differentiation using Pearson correlation analysis. We found that the protein amount of RAB37 was associated with TIMP1 secretion during adipogenic differentiation (Figure 3(e)). The presence of interactions between RAB37 and TIMP1 was detected by PLA on day 5 of adipogenic differentiation. As compared to control hADSCs, increased PLA signals representing pairs of RAB37 and TIMP1 protein complex were observed in hADSCs after 5 days of adipogenic induction (Figure 3(f)).

### 3.4. Knockdown of TIMP1 Impeded RAB37-Mediated Adipogenic Differentiation

**Figure 4:**
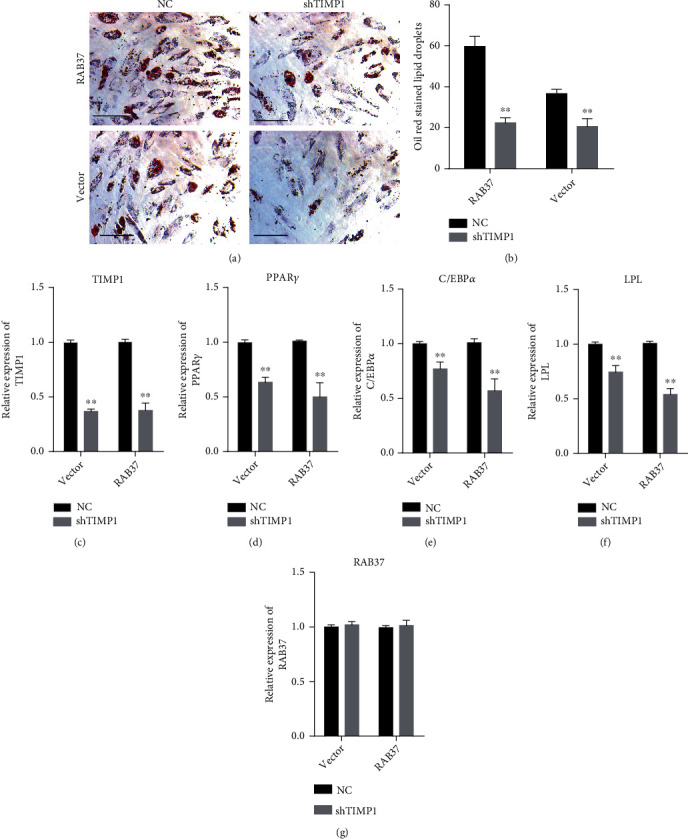
Knockdown of TIMP1 compromised RAB37-mediated adipogenic differentiation. The lentivirus expressing shRNA targeting TIMP1 (lv-shTIMP1) was used to inhibit endogenous expression of TIMP1 in hADSCs-RAB37 and hADSCs-vector. Lentivirus expressing a scramble RNA (NC) was used as control. Adipogenic differentiation was evaluated by Oil Red O staining (a) (scale bar = 300 *μ*m). Oil Red O-stained hADSCs in six random views were counted (b), *n* = 6. The expression of TIMP1 (c), PPAR*γ* (d), C/EBP*α* (e), and LPL (f) were examined by qRT-PCR, *n* = 3. Data are presented as mean ± SD. ^∗∗^*p* < 0.01; NC vs. shTIMP1.

We further explored the involvement of TIMP1 in RAB37-mediated adipogenic differentiation of hADSCs. The endogenous expression of TIMP1 in hADSCs-RAB37 and hADSCs-vector was inhibited by lentivirus expressing shRNA targeting TIMP1 (lv-shTIMP1). Lentivirus expressing scramble RNA (NC) served as control for lv-shTIMP1. Oil Red O staining showed that shTIMP1 decreased lipid droplets in both hADSCs-RAB37 and hADSCs-vector, as compared to NC (Figures 4(a) and 4(b)). qRT-PCR showed that shTIMP1 decreased the expression of TIMP1, PPAR*γ*, C/EBP*α*, and LPL but did not affect the expression of RAB37 (Figures 4(c)–4(g)).

### 3.5. Involvement of the TIMP1/CD63/Integrin *β*1 Axis in RAB37-Mediated Adipogenic Differentiation

**Figure 5:**
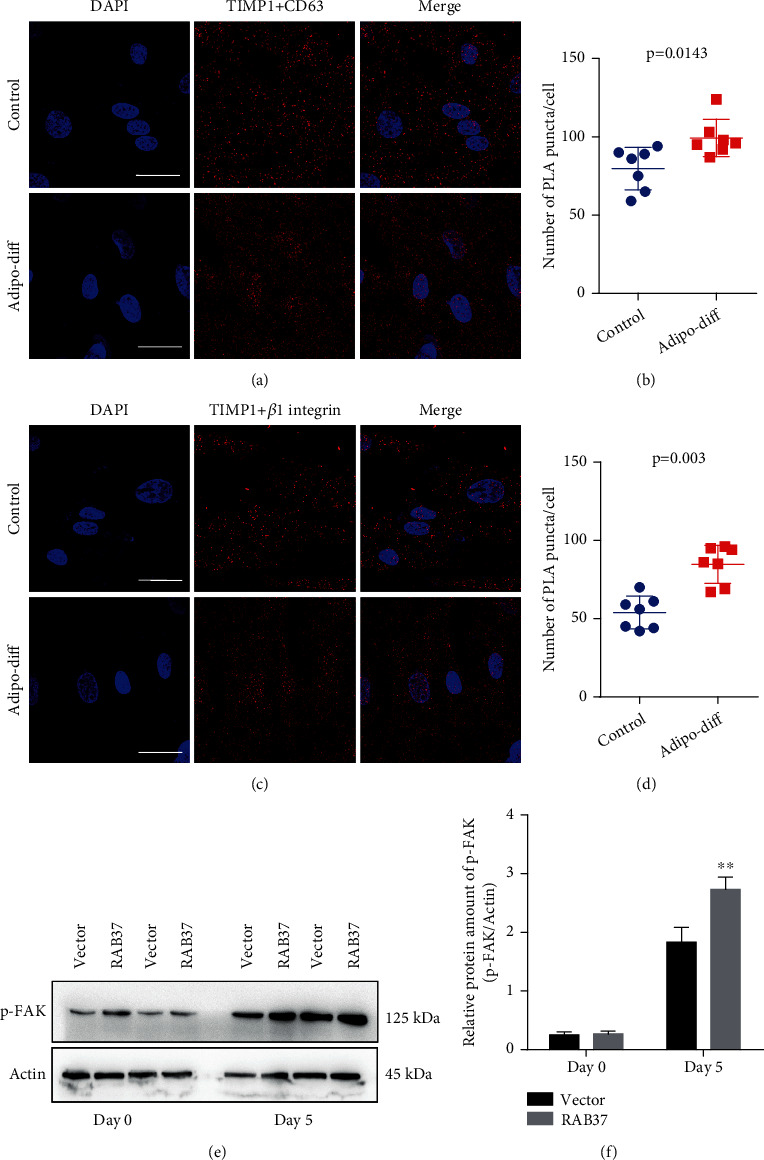
TIMP1 activated integrin *β*1 signaling in RAB37-mediated adipogenic differentiation. (a) hADSCs were subjected to induction of adipogenic differentiation for 5 days. PLA was performed to determine the interaction between TIMP1 and CD63. (b) Quantitative analysis of PLA assay of TIMP1 and CD63, *n* = 8. (c) PLA was performed to determine the interaction between TIMP1 and integrin *β*1. (d) Quantitative analysis of PLA assay of TIMP1 and integrin *β*1, *n* = 8. (e) Western blotting was used to evaluate the accumulation of phosphorylated FAK in hADSCs-RAB37 and hADSCs-vector after induction of adipogenic differentiation. (f) Quantitative analysis of phosphorylated FAK, *n* = 3. Data are presented as means ± SD. ^∗∗^*p* < 0.01; vector vs. RAB37. Scale bar for (a) and (c) = 50 *μ*m.

Previous studies have demonstrated that TIMP1 is involved in many cellular activities by activating integrin *β*1 signaling, which requires the binding of TIMP1 to membrane receptor CD63 and integrin *β*1 (22, 23). We examined the binding of TIMP1 to membrane receptor CD63 and integrin *β*1 in hADSCs during adipogenic differentiation using PLA. Undifferentiated hADSCs were used as control. PLA showed that TIMP1 could bind to both CD63 and integrin *β*1 in both undifferentiated hADSCs and adipogenic differentiated hADSCs. Increased PLA signals of TIMP1 and CD63 were observed in hADSCs after induction of adipogenic differentiation, as compared to the control (Figures 5(a) and 5(b)). Similar augmentation by adipogenic differentiation was also observed in PLA signals of TIMP1 and integrin *β*1 (Figures 5(c) and 5(d)). As a parallel control, the interaction between TIMP1 and CD63/integrin *β*1 was also examined in Human Umbilical Vein Endothelial Cells (HUVECs) (Figure S2).

FAK is an important protein kinase of the integrin *β*1 signaling pathway (22, 23). We found increased S397 phosphorylation of FAK in hADSCs after induction of adipogenic differentiation, as compared to undifferentiated hADSCs. Exogenous expression of RAB37 increased the expression S397 phosphorylation of FAK in hADSCs after induction of adipogenic differentiation (Figures 5(e) and 5(f)).

### 3.6. Knockdown of CD63 Compromised RAB37-Mediated Adipogenic Differentiation

**Figure 6:**
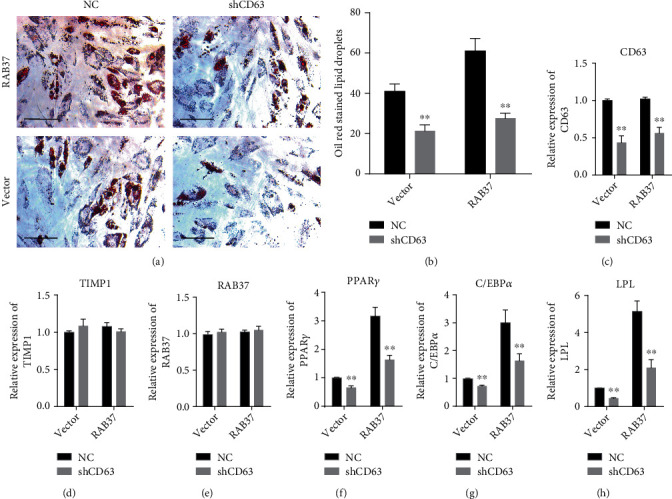
Knockdown of CD63 compromised RAB37-mediated proliferation and adipogenic differentiation. The lentivirus expressing shRNA targeting CD63 (shCD63) was used to inhibit endogenous expression of CD63 in RAB37-ADSCs. Lentivirus expressing a scramble RNA was used as control. Adipogenic differentiation was evaluated by Oil Red O staining (a); scale bar = 300 *μ*m. Oil Red O-stained hADSCs in six random views were counted (b), *n* = 6. The expression of TIMP1 (c), PPAR*γ* (d), C/EBP*α* (e), and LPL (f) was examined by qRT-PCR, *n* = 3. Data are presented as mean ± SD. ^∗∗^*p* < 0.01; lv-scramble vs. lv-shCD63.

We further explored the involvement of membrane receptor CD63 in RAB37-mediated adipogenic differentiation. Lentivirus expressing shRNA targeting CD63 (lv-shCD63) was used to inhibit endogenous expression of CD63 in hADSCs-RAB37 and hADSCs-vector. Lentivirus expressing a scramble RNA (NC) was used as control. Decreased Oil Red O-stained lipid droplets were observed in lv-shCD63 infected hADSCs-RAB37 (Figures 6(a) and 6(b)). qRT-PCR showed that shCD63 decreased the expression of CD63, PPAR*γ*, C/EBP*α*, and LPL but did not affect the expression of TIMP1 or RAB37 (Figures 6(c)–6(h)).

### 3.7. FAK Inhibitor PF-573228 Compromised RAB37-Mediated Adipogenic Differentiation

**Figure 7:**
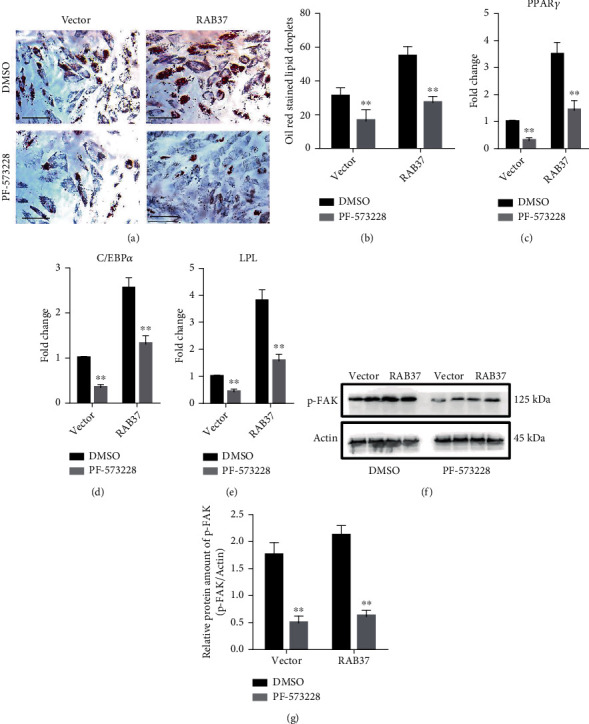
An inhibitor of FAK impeded RAB37-mediated adipogenic differentiation RAB37-hADSCs was pretreated with PF-573228, an inhibitor of FAK, and then subjected to induction of adipogenic differentiation for 5 days. DMSO was used as control for PF-573228. Adipogenic differentiation was evaluated by Oil Red O staining (a); scale bar = 300 *μ*m. Oil Red O-stained hADSCs in six random views were counted (b), *n* = 6. The expression of PPAR*γ* (c), C/EBP*α* (d), and LPL (e) was examined by qRT-PCR, *n* = 3. The accumulations of phosphorylated FAK in hADSCs were examined by western blotting (f). (g) Quantitative analysis of phosphorylated FAK, *n* = 3. Data are presented as mean ± SD. ^∗∗^*p* < 0.01; DMSO vs. PF-573228.

We also explored the involvement of FAK in RAB37-mediated adipogenic differentiation. A chemical inhibitor PF-573228 was used to inhibit the activation of FAK in hADSCs-RAB37 and hADSCs-vector. DMSO was used as control for PF-573228. Decreased Oil Red O-stained lipid droplets were observed in PF-573228 treated hADSCs-RAB37 and hADSCs-vector (Figures 7(a) and 7(b)). qRT-PCR showed that PF-573228 decreased the expression of CD63, PPAR*γ*, C/EBP*α*, and LPL (Figures 7(c)–7(e)). The efficiency of PF-573228 was verified by the decreased level of Tyr397-phosphorylated FAK (Figures 7(f) and 7(g)).

## 4. Discussion

In the current study, we report that RAB37 promotes adipogenic differentiation of hADSCs by regulating the TIMP1/CD63/integrin *β*1 signaling pathway. We found that the mRNA level of RAB37 fluctuates during adipogenic differentiation. Gain-of-function and loss-of-function experiments demonstrated the regulatory role of RAB37. Further exploration on the molecular mechanism identified TIMP1 as an upregulated cytokine associated with RAB37-mediated adipogenic differentiation. In addition, RAB37 can promote the secretion of TIMP1 by hADSCs. RAB37-mediated adipogenic differentiation requires the TIMP1/CD63/integrin *β*1 axis.

One of the important finding is the revealing of the increased accumulation of RAB37 from the early stage of adipogenic differentiation, and increased accumulation of RAB37 may facilitate adipogenic differentiation. We showed that upregulation of RAB37 enhanced, while its downregulation inhibited adipogenic differentiation, as determined by Oil Red O staining and measurement of the expression of PPAR*γ*, C/EBP*α*, and LPL. Our findings provide novel insight into the physiological significance of RAB37. The expression level of RAB37 changes according to different physiological and pathological conditions. RAB37 can be stimulated by LPS and inhibited by proinflammatory cytokines, fatty acids, and oxidized low-density lipoprotein (27, 28).

The other finding is that RAB37 promotes adipogenic differentiation by regulating the secretion of TIMP1. Our data showed increased accumulation of TIMP1 in the supernatant of hADSCs-RAB37 compared with hADSCs-vector, and the concentration of TIMP1 reached the peak after 5 days of adipogenic induction. Moreover, the protein amount of RAB37 was correlated with TIMP1 secretion during adipogenic differentiation. These data demonstrate the regulatory role of RAB37 in the secretion of TIMP1 during adipogenic differentiation, which is consistent with previous finding in lung cancer that RAB37 inhibits the migration of lung cancer cells by regulating the secretion of TIMP1 (15). RAB37-facilitated TIMP1 secretion may occur in several types of cells, with different physiological or pathological significance under different context. RAB37 plays a positive role in adipogenic differentiation of hADSCs, but a negative role in lung cancer cell migration.

We also provide evidence for the involvement of the TIMP1/CD63/integrin *β*1 axis in RAB37-mediated adipogenic differentiation. Our data showed that TIMP1 binds to both CD63 and integrin *β*1 in hADSCs during adipogenic differentiation. The interactions between TIMP1 and these two receptors occur throughout the early stage of adipogenic differentiation. Knockdown of CD63 impeded RAB37-mediated adipogenic differentiation. Moreover, we also found the activation of FAK, a downstream protein kinase of integrin *β*1 signaling, in RAB37-mediated adipogenic differentiation. Increased Tyr397 phosphorylation of FAK was observed during adipogenic differentiation, suggesting that activation of FAK can be inducted by adipogenic differentiation. PF-573228, an inhibitor of FAK, compromised RAB37-mediated adipogenic differentiation. These results indicate that the TIMP1/CD63/integrin *β*1 axis is required for the RAB37-mediated adipogenic differentiation.

However, there are still several issues that need to be resolved. One issue is whether TIMP1's involvement in adipogenic differentiation requires binding to both CD63 and integrin *β*1, or only one of the receptors. Our data only suggest that inhibition of CD63 or blockade of integrin *β*1 signaling inhibits adipogenic differentiation. Further study is required to determine which one is the dominant receptor in TIMP1's involvement in adipogenic differentiation. In addition, this study only provides in vitro evidence for the RAB37-mediated adipogenic differentiation of hADSCs. In the future study, we will explore the mechanism of RAB37-mediated adipogenic differentiation in nude mice.

## Data Availability

All data included in this study are available from the corresponding author by reasonable request.

## Supplementary Materials

Supplementary Materials 1: characterization of hADSCs. The multipotency of hADSCs was confirmed by adipogenic, osteogenic, and endothelial differentiation. Adipogenic and osteogenic differentiation was evaluated by Oil Red O staining (Sigma-Aldrich, St. Louis, MO, USA) for lipid droplets and Alizarin red staining (Sigma-Aldrich, St. Louis, MO, USA) for calcium deposit. Endothelial differentiation was measured by tube formation assays. 2. PLA assay in HUVECs. The interaction between TIMP1 and RAB37, TIMP1 and CD63, and TIMP1 and *β*1 integrin in HUVECs was examined by PLA, as a parallel control of hADSCs. Briefly, hADSCs were fixed with 4% paraformaldehyde, incubated with PBS containing 0.1% Triton X-100, and blocked with Blocking Solution for 60 min. hADSCs were incubated with primary antibodies at 4°C overnight. Unbound antibodies were removed by washing in PBS-T, and the cells were then incubated with oligonucleotide-conjugated secondary antibodies (PLA Probes) PLUS and MINUS for 1 h at 37°C. Subsequently, cells were washed and incubated with a ligation mixture (Detection Reagent Red) for 30 min at 37°C. Cells were washed and incubated with an amplification mixture (Detection Reagent Red) for 100 min at 37°C. After amplification, cells were washed and rinsed in distilled water. The nuclei were stained with DAPI. Cells were visualized using a FV3000 Olympus laser scanning confocal microscope with a 100x objective. Rolling circle products (RCPs) were quantified using the Duolink Image Tool. For clear visualization of RCPs, the PLA images were processed with ImageJ software using a maximum filter (3 pixels) for the PLA channel.
