# Supplementary material for: RAB37 Promotes Adipogenic Differentiation of hADSCs via the TIMP1/CD63/Integrin Signaling Pathway
Source: Stem Cells Int. 2021 Nov 23;2021:8297063. doi: 10.1155/2021/8297063 (PMC8632429; doi:10.1155/2021/8297063)
Supplement: Supplementary Materials — 1: characterization of hADSCs. The multipotency of hADSCs was confirmed by adipogenic, osteogenic, and endothelial differentiation. Adipogenic and osteogenic differentiation was evaluated by Oil Red O staining (Sigma-Aldrich, St. Louis, MO, USA) for lipid droplets and Alizarin red staining (Sigma-Aldrich, St. Louis, MO, USA) for calcium deposit. Endothelial differentiation was measured by tube formation assays. 2. PLA assay in HUVECs. The interaction between TIMP1 and RAB37, TIMP1 and CD63, and TIMP1 and β1 integrin in HUVECs was examined by PLA, as a parallel control of hADSCs. Briefly, hADSCs were fixed with 4% paraformaldehyde, incubated with PBS containing 0.1% Triton X-100, and blocked with Blocking Solution for 60 min. hADSCs were incubated with primary antibodies at 4°C overnight. Unbound antibodies were removed by washing in PBS-T, and the cells were then incubated with oligonucleotide-conjugated secondary antibodies (PLA Probes) PLUS and MINUS for 1 h at 37°C. Subsequently, cells were washed and incubated with a ligation mixture (Detection Reagent Red) for 30 min at 37°C. Cells were washed and incubated with an amplification mixture (Detection Reagent Red) for 100 min at 37°C. After amplification, cells were washed and rinsed in distilled water. The nuclei were stained with DAPI. Cells were visualized using a FV3000 Olympus laser scanning confocal microscope with a 100x objective. Rolling circle products (RCPs) were quantified using the Duolink Image Tool. For clear visualization of RCPs, the PLA images were processed with ImageJ software using a maximum filter (3 pixels) for the PLA channel. [file 8297063.f1.docx]

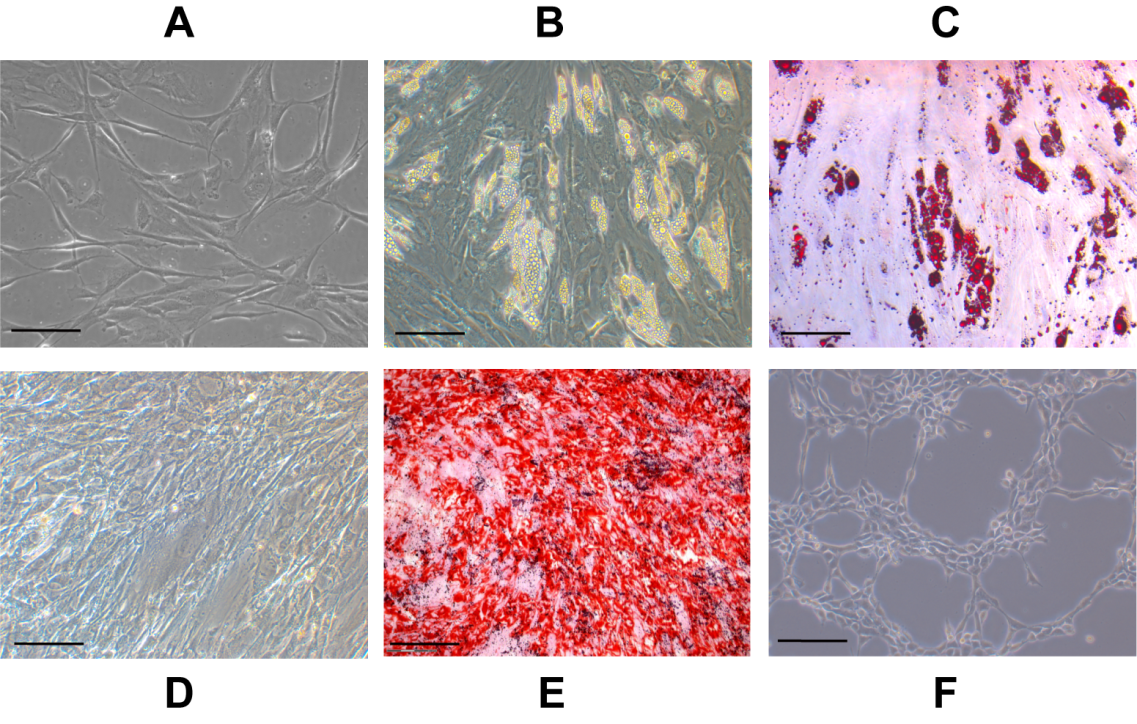


Figure S1 characterization of ADSCs

A, representative image of hADSCs. B, representative image of adipogenic differentiating hADSCs. C, representative image of oil red staining in hADSCs after adipogenic induction. D, representative image of osteogenic differentiating hADSCs. E, representative image of Alizarin red staining in hADSCs after osteogenic induction. F, representative image of tube formation assay in hADSCs after endothelial induction. Scale bar=300μm


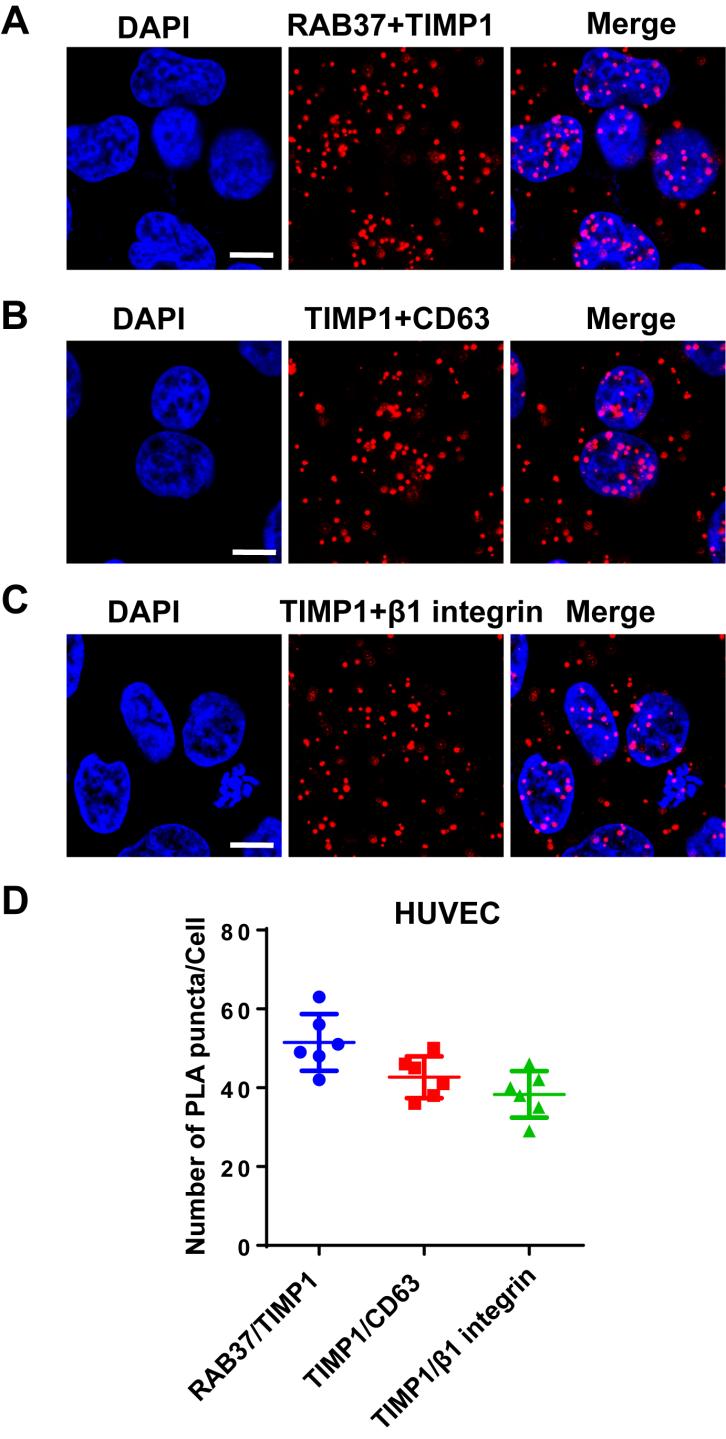


Figure S2 interaction between TIMP1 and RAB37(A), TIMP1 and CD63 (B), TIMP1 and β1 integrin (C) in Human Umbilical Vein Endothelial Cells (HUVECs) were examined by PLA. PLA puncta in six random views were counted (B). Scatter diagram represented means±SD. Scale bar for A, B and C=50μm

Figure S3 Original image of figure 1E


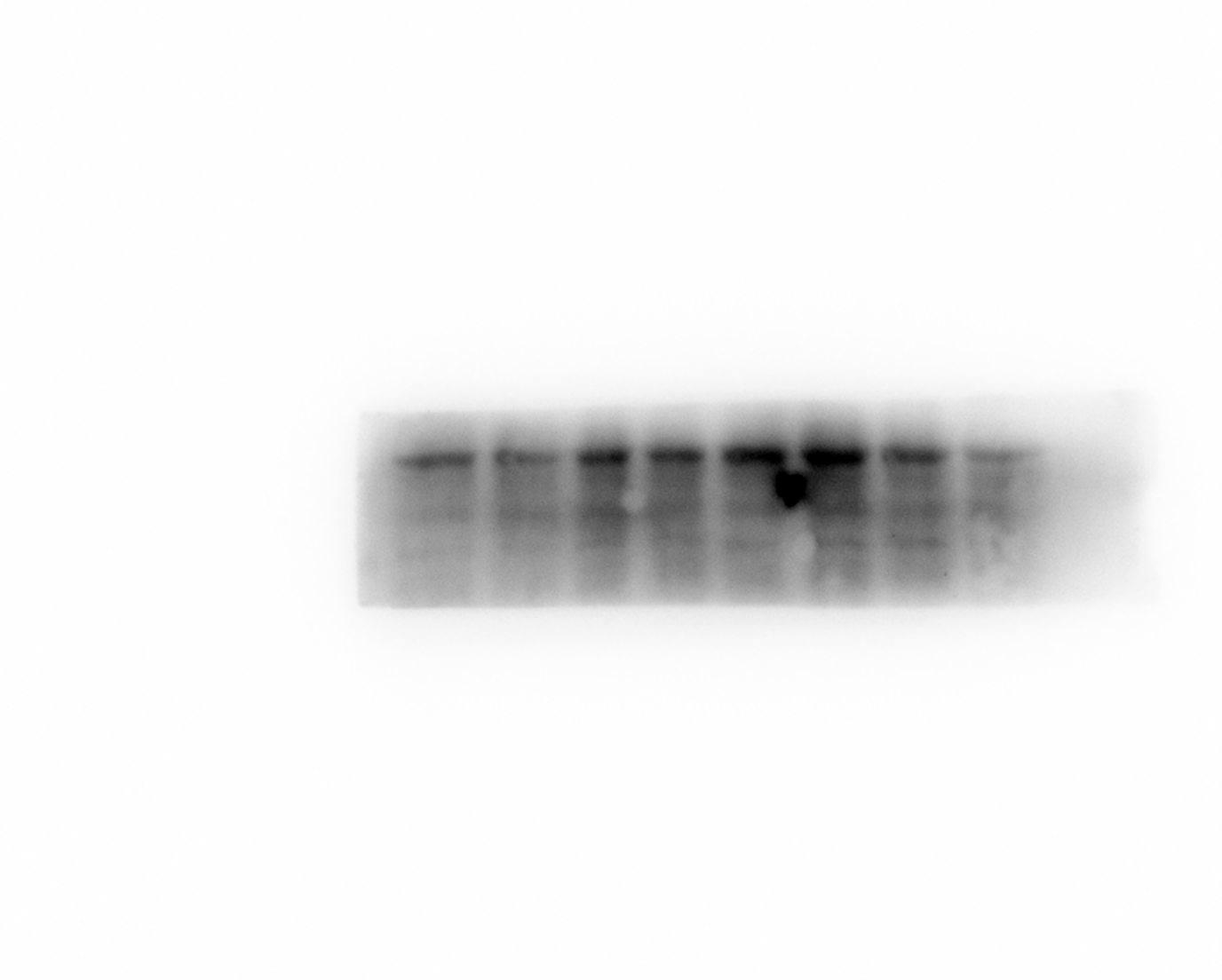

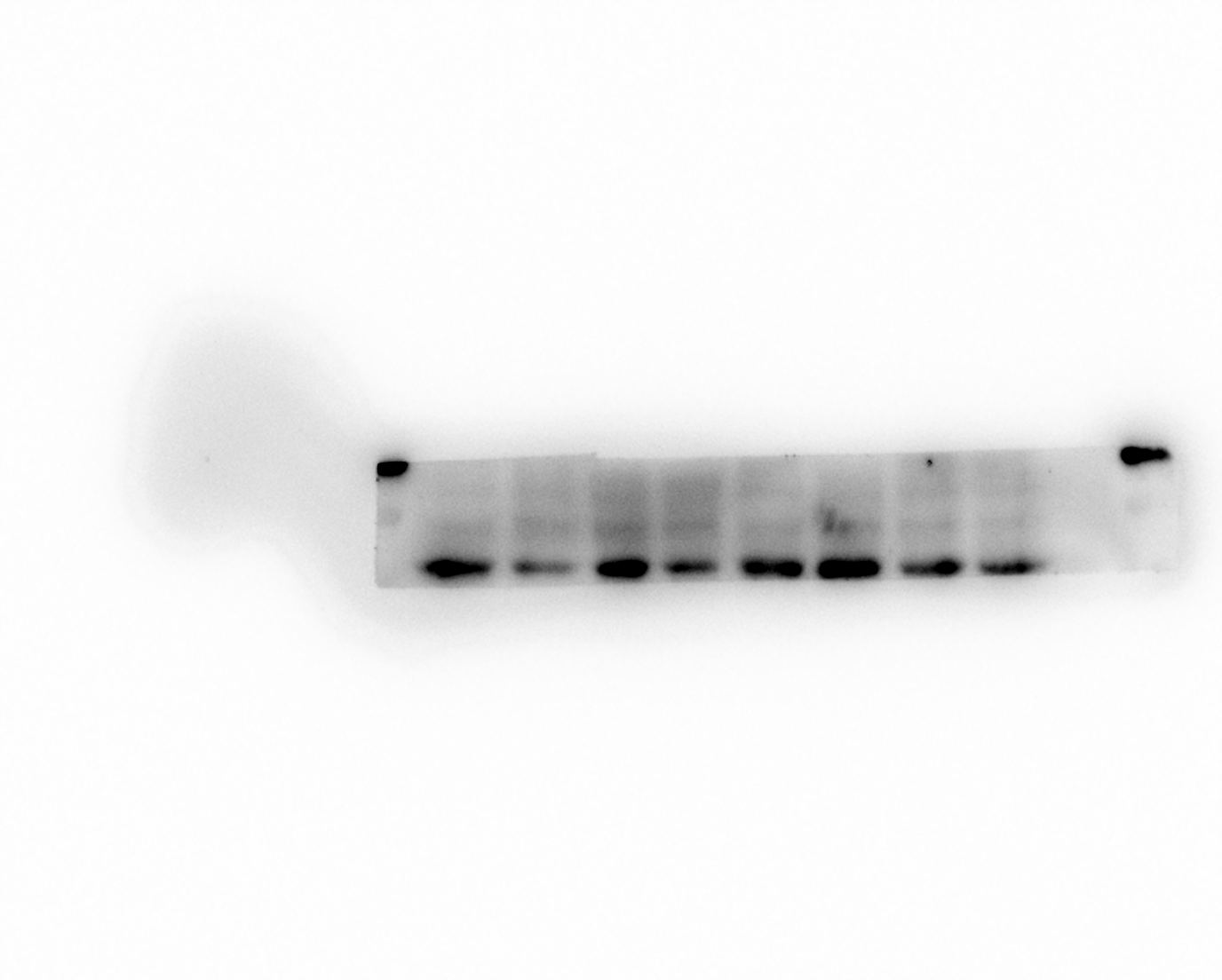


**C/EBPα**

RAB37


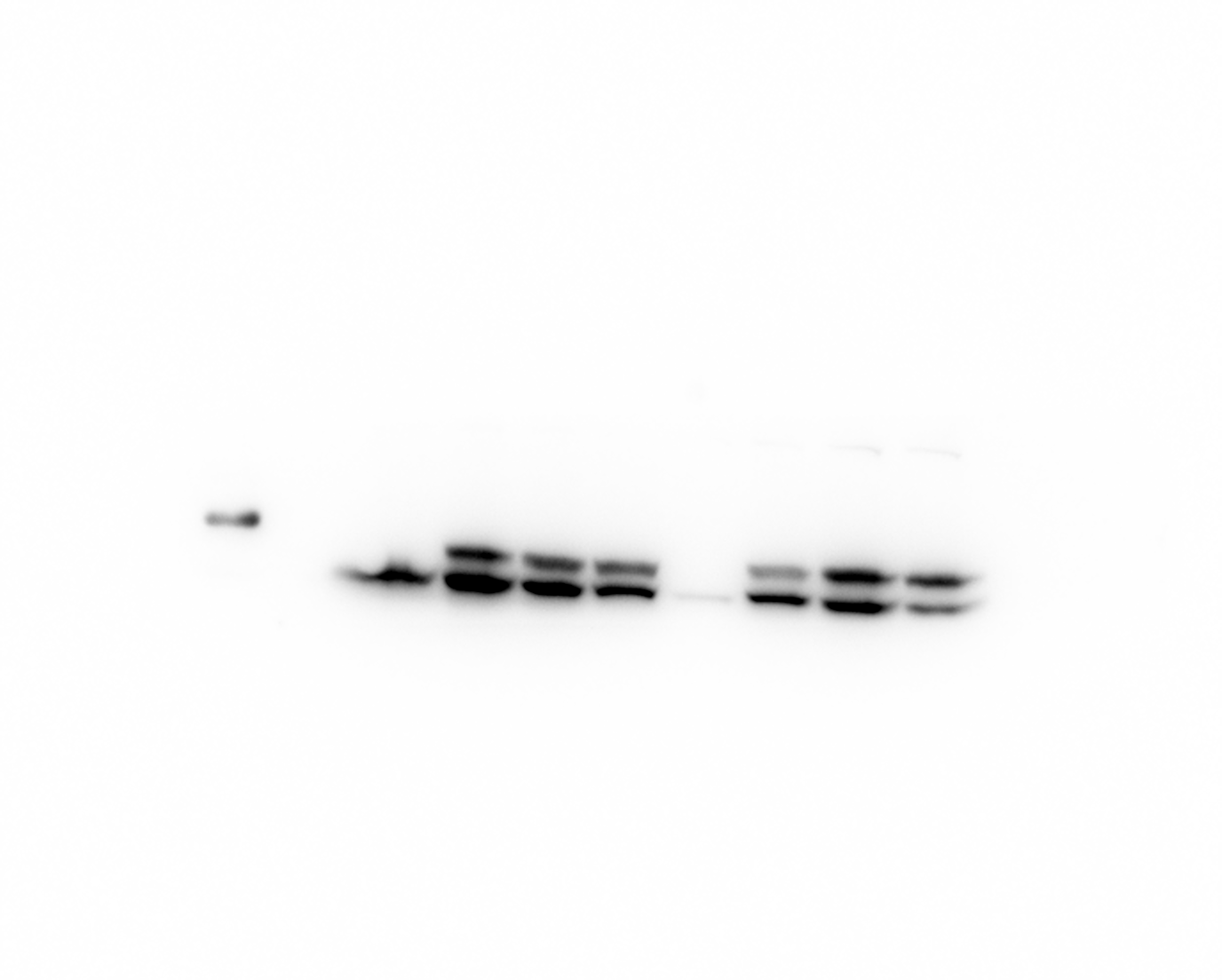

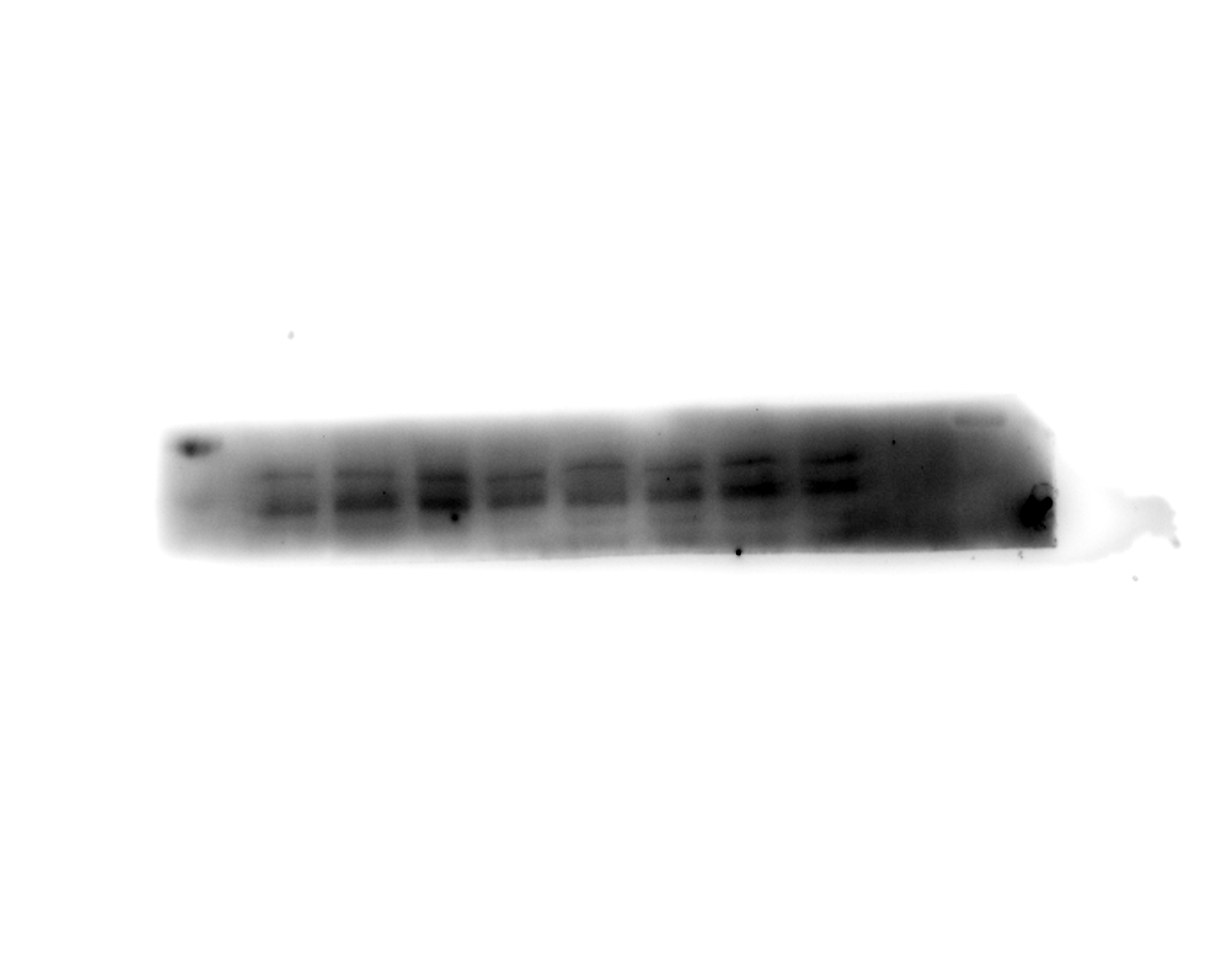


LPL

**PPARγ**


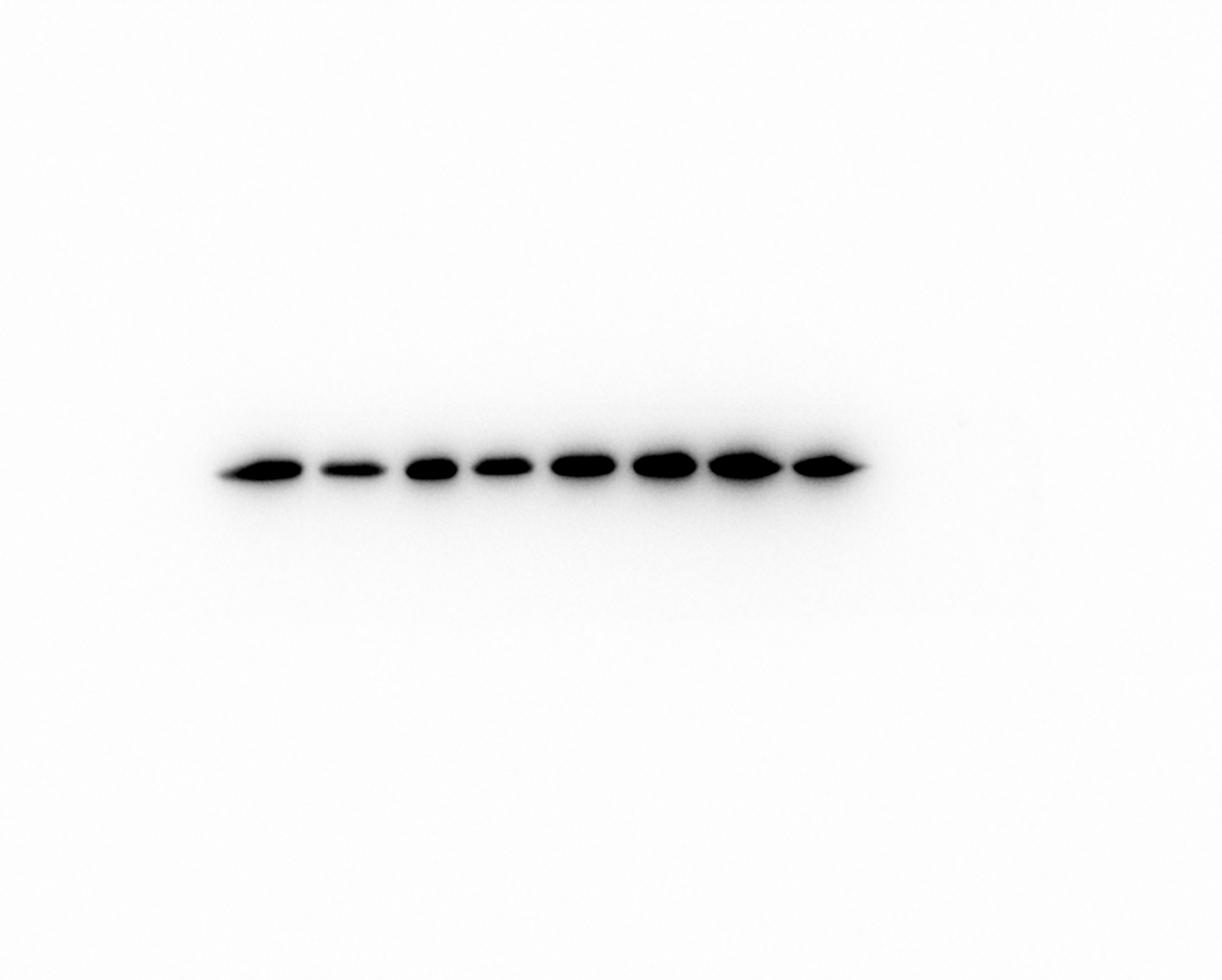


GAPDH

**Figure S4 Original image of figure 3D RAB37 and GAPDH**


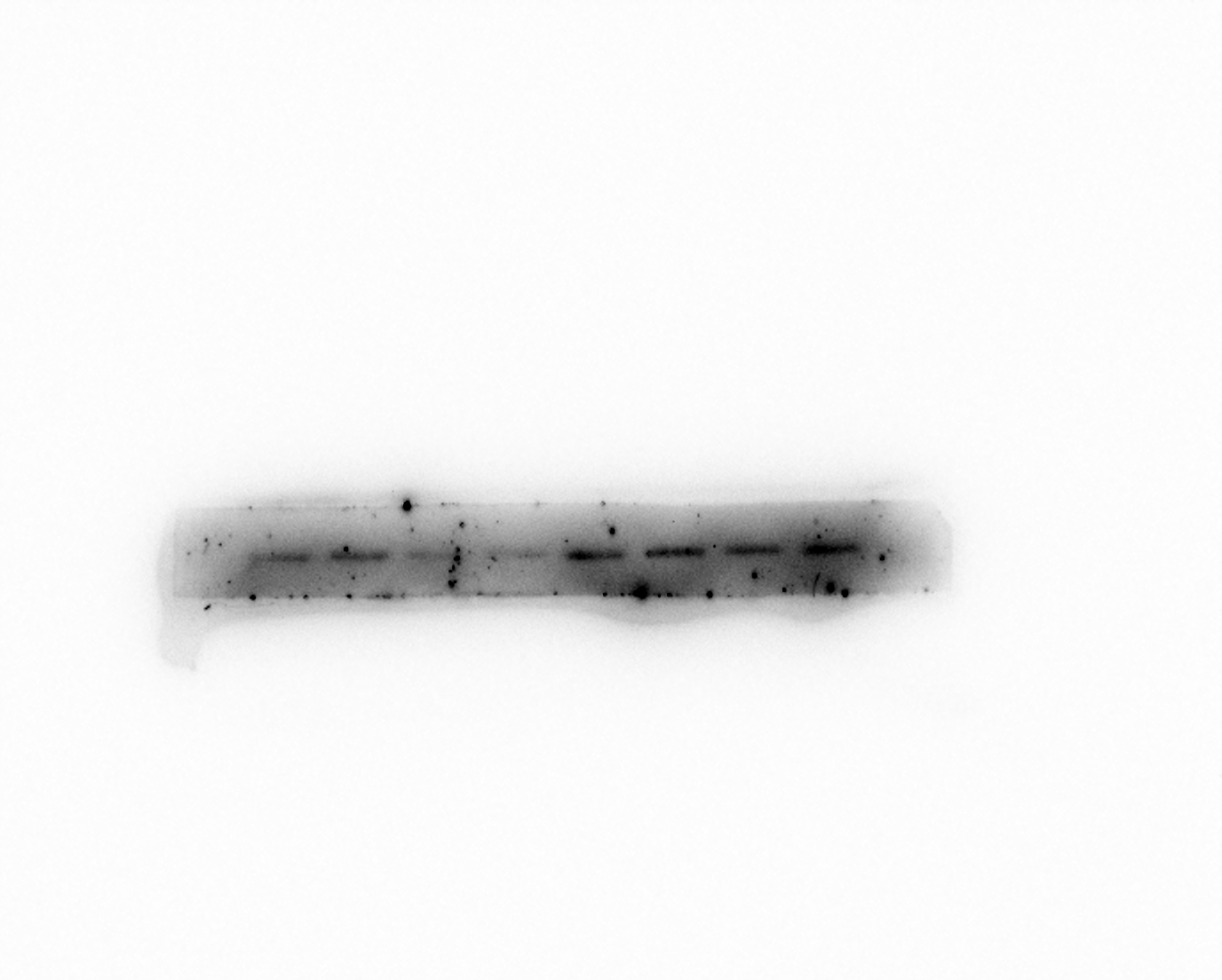

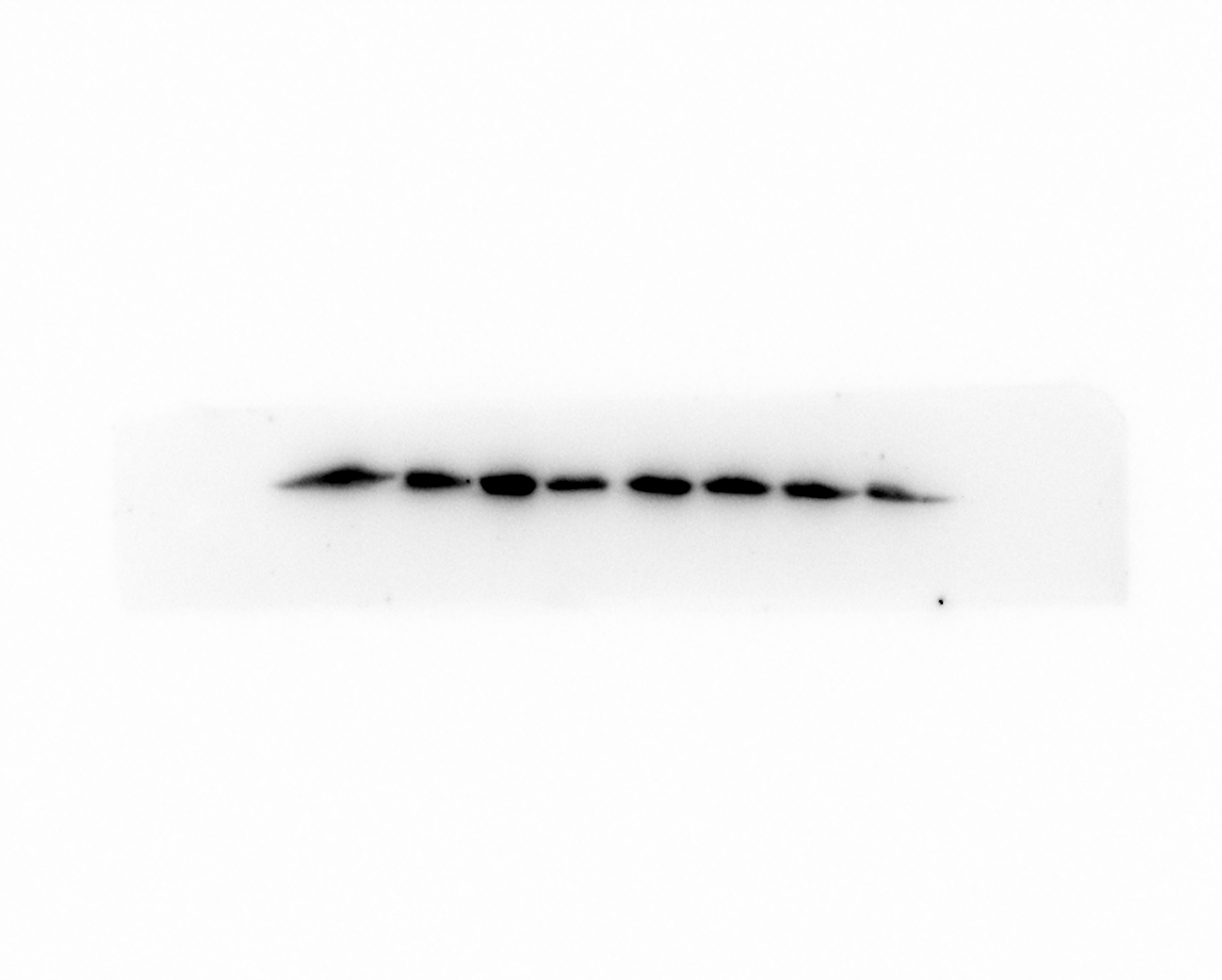


Figure S5 Original image of figure 5E


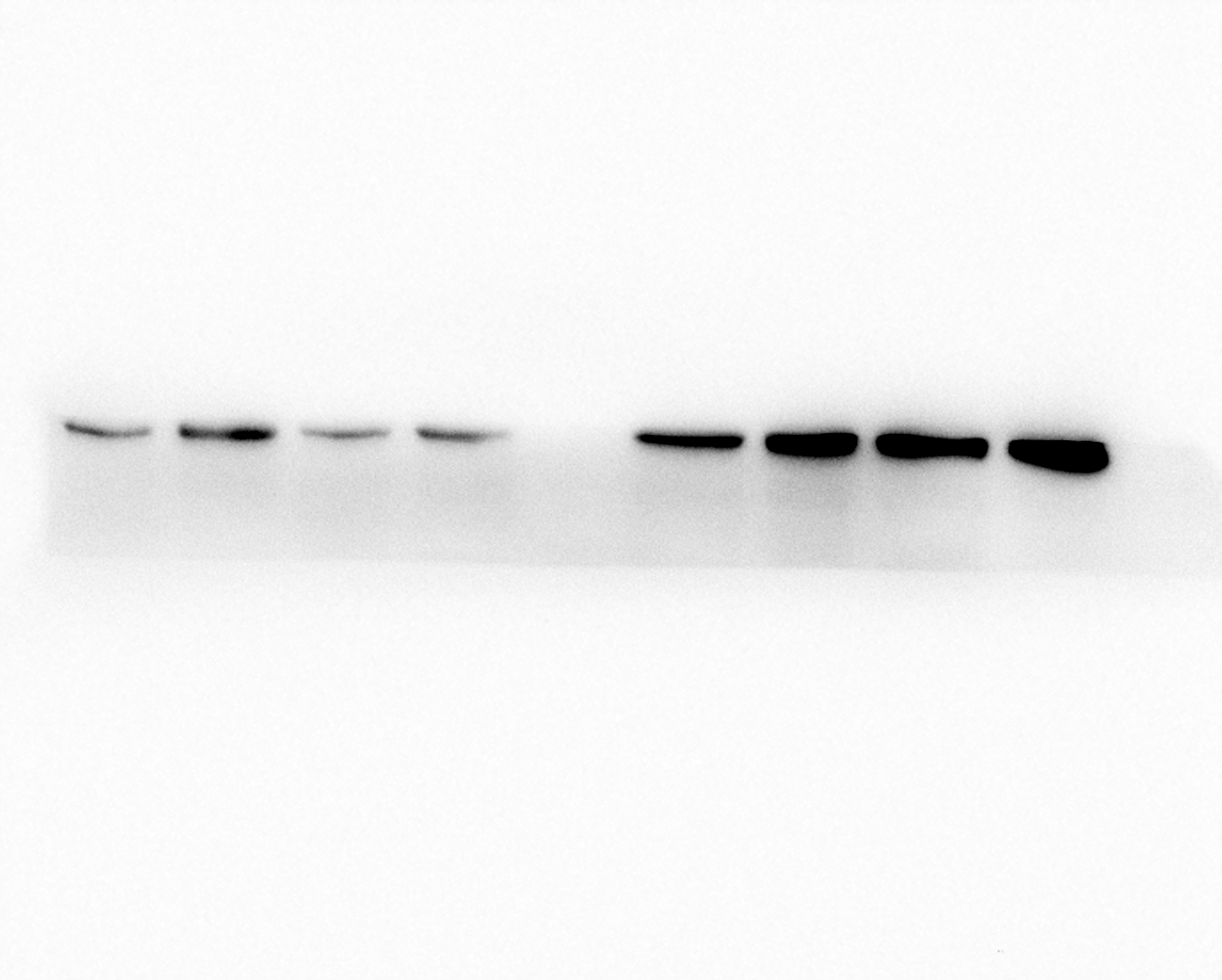


**p-FAK**


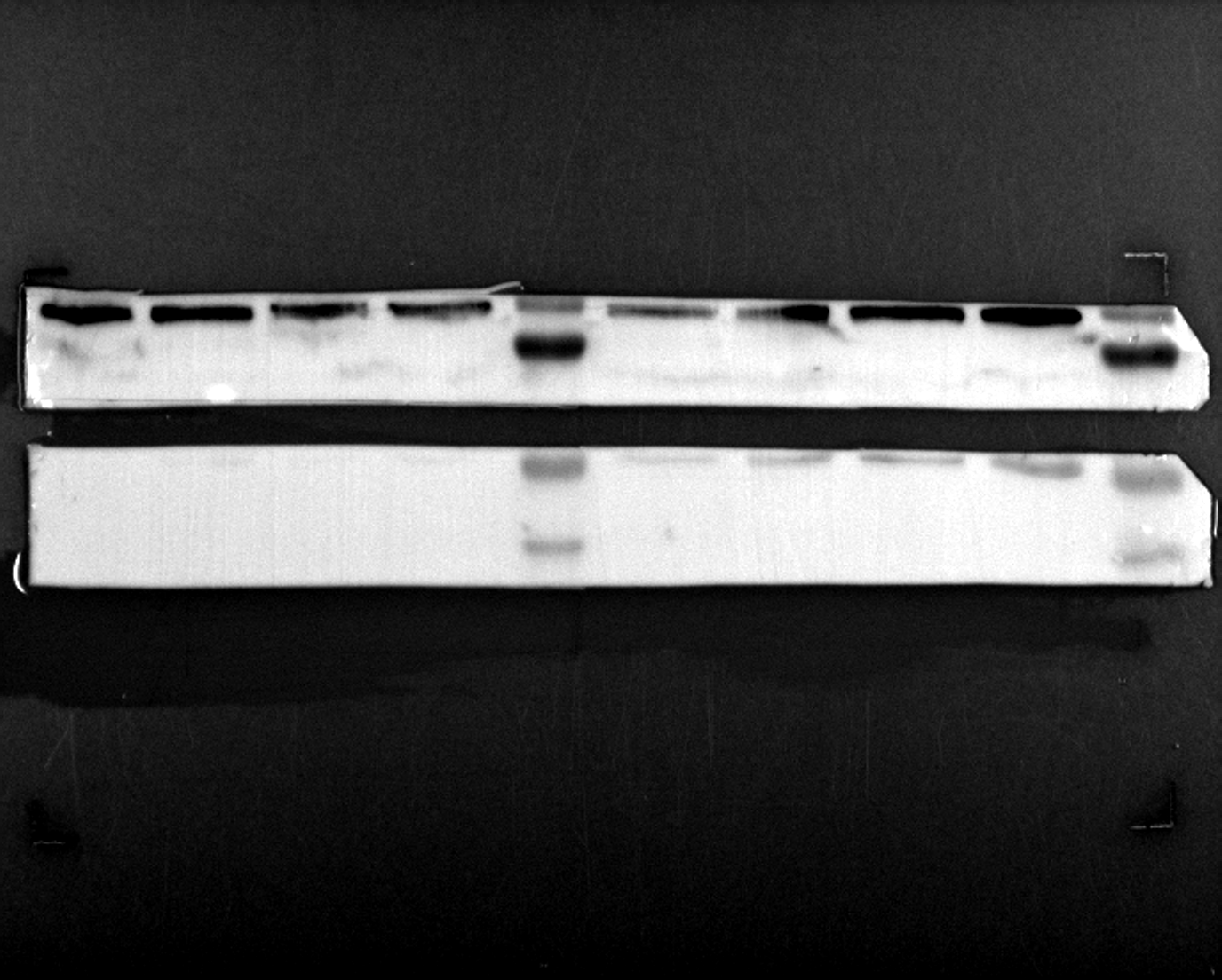


**Image with protein ladder**

**Actin**


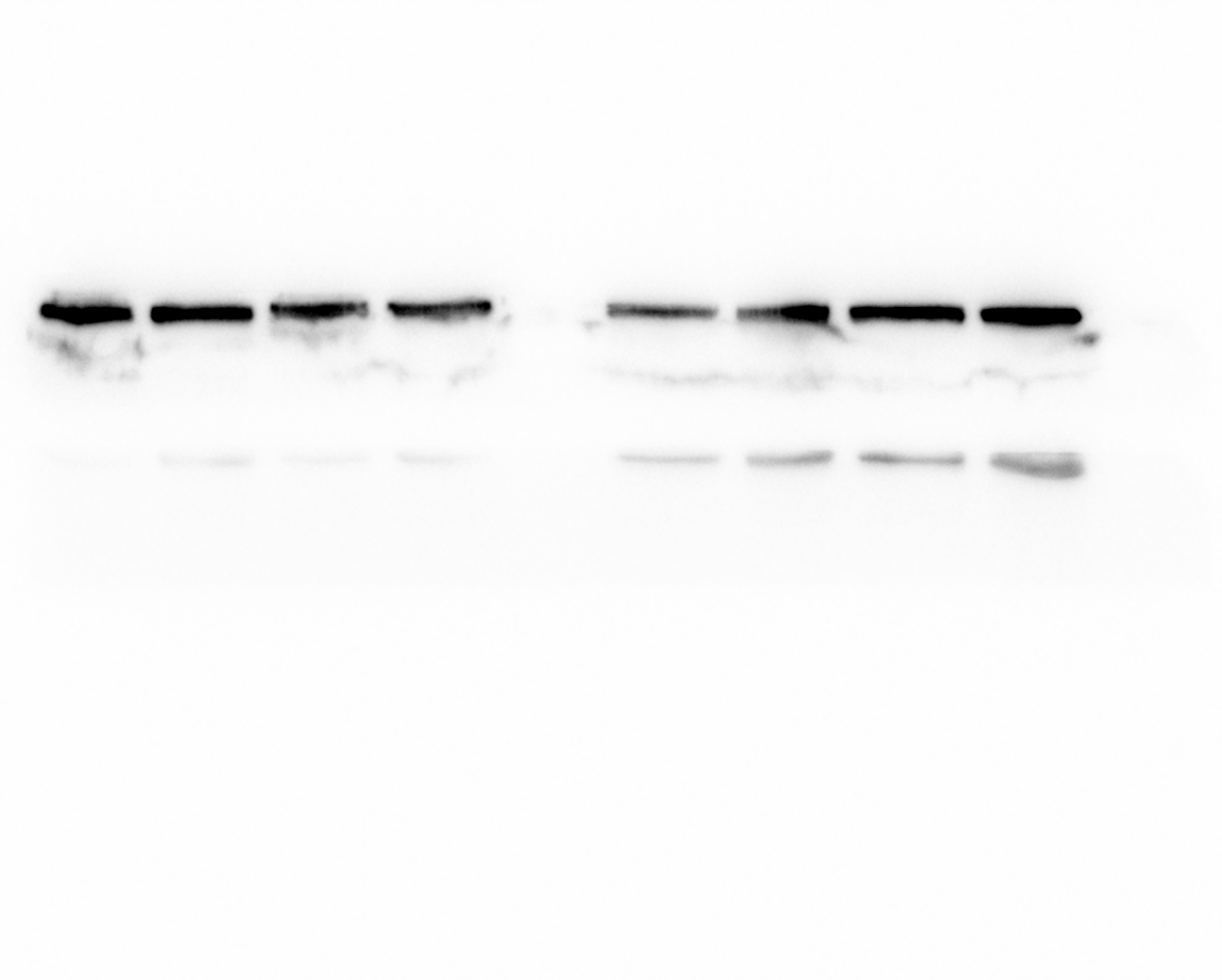


**Actin**

**Image without protein ladder**

Figure S6 Original image of figure 7F p-FAK and actin


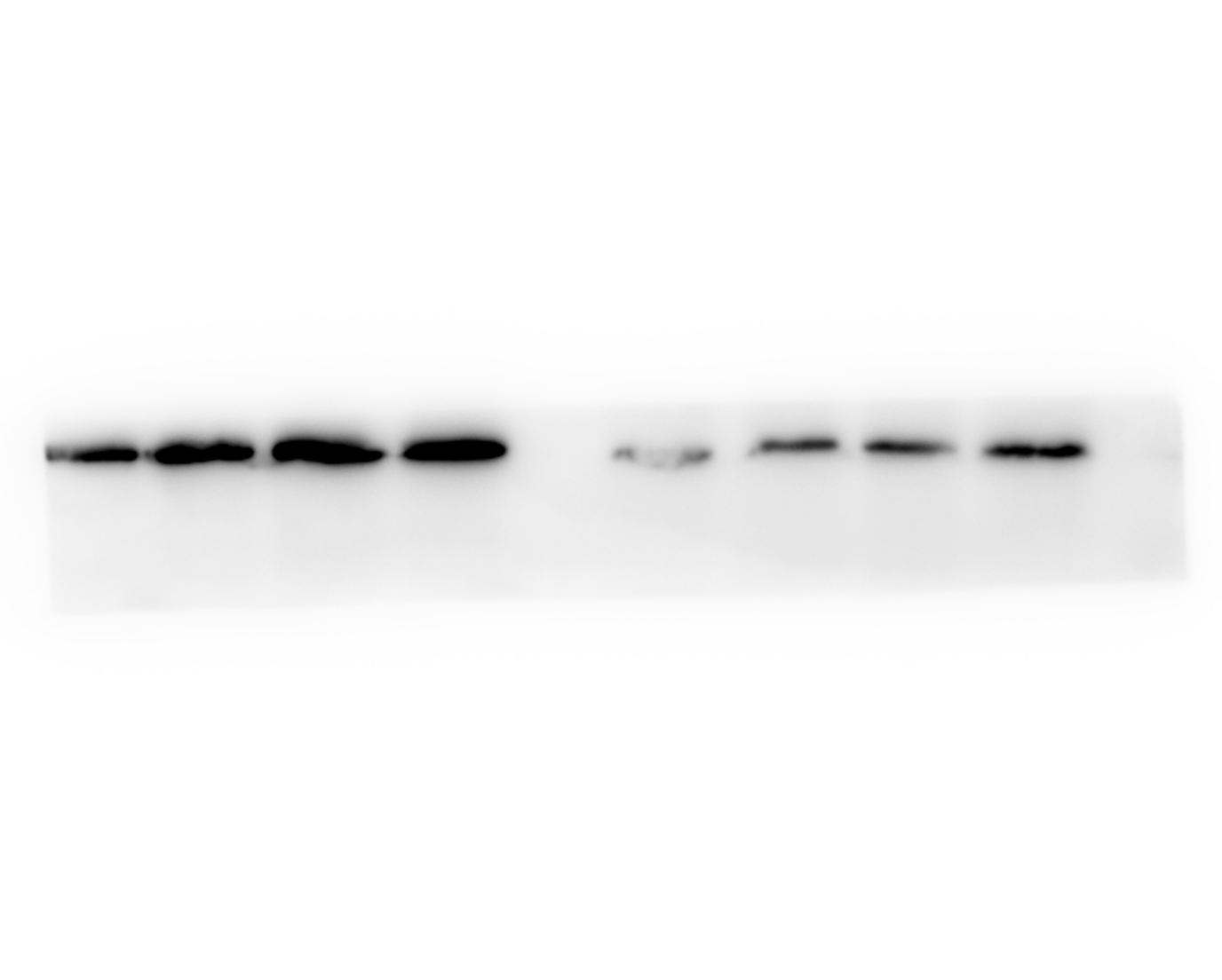


**p-FAK**


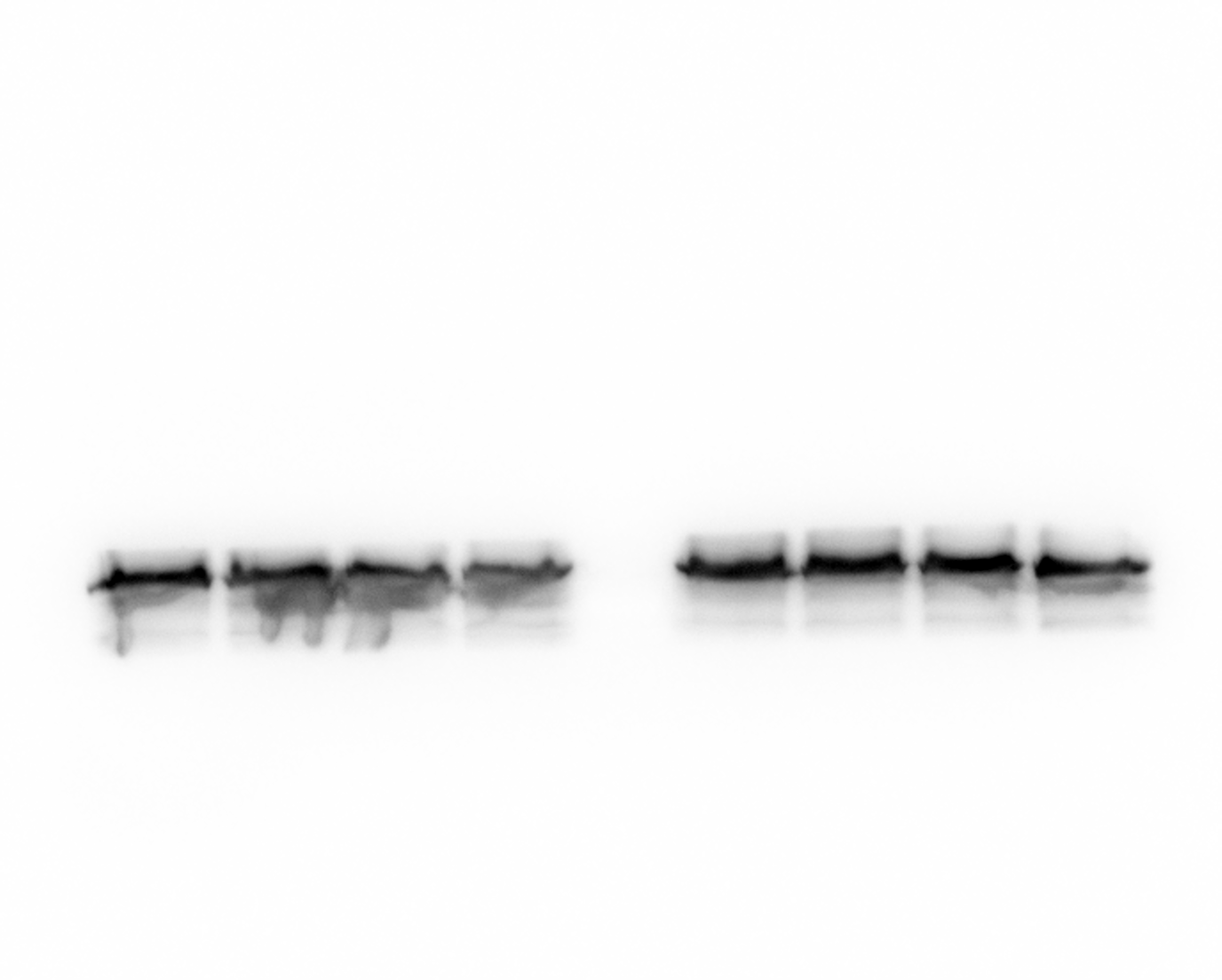


**Actin**

**Table S1 QPCR primers**

| ID | Sequence |  |
| --- | --- | --- |
| LPL Forward | CTGCTGGCATTGCAGGAAGTCT |  |
| LPL Reverse | CATCAGGAGAAAGACGACTCGG |  |
| PPARg Forward | AGCCTGCGAAAGCCTTTTGGTG |  |
| PPARg Reverse | GGCTTCACATTCAGCAAACCTGG |  |
| C/EBPa Forward | AGGAGGATGAAGCCAAGCAGCT |  |
| C/EBPa Reverse | AGTGCGCGATCTGGAACTGCAG |  |
| RAB37 Forward | GCAAGGTGATGCTTCTGGGAGA |  |
| RAB37 Reverse | CCTTGTTCCTGAAGTCTATGCCG |  |
| TIMP1 Forward | GGAGAGTGTCTGCGGATACTTC |  |
| TIMP1 Reverse | GCAGGTAGTGATGTGCAAGAGTC |  |
| CD63 Forward | CAACCACACTGCTTCGATCCTG |  |
| CD63 Reverse | GACTCGGTTCTTCGACATGGAAG |  |
| GAPDH Forward | GTCTCCTCTGACTTCAACAGCG |  |
| GAPDH Reverse | ACCACCCTGTTGCTGTAGCCAA |  |

**Table S2 Primary antibodies for PLA assay**

| antibodies |  | Brand | Catalog NO. | Delution |
| --- | --- | --- | --- | --- |
| anti-RAB37 antibody | rabbit | abcam | ab233114 | 1:100 |
| anti-TIMP1 antibody | mouse | Santa Cruz | Sc-365905 | 1:50 |
| anti-TIMP1 antibody | rabbit | abcam | ab211926 | 1:100 |
| anti-CD63 antibody | mouse | abcam | ab1318 | 1:100 |
| anti-integrinβ1 antibody | mouse | abcam | ab30394 | 1:100 |
